# Association Between Patient Satisfaction With Their Patient-Physician Relationship and Completion of Bariatric Surgery by Race and Ethnicity Among US Adults

**DOI:** 10.1001/jamanetworkopen.2022.47431

**Published:** 2022-12-19

**Authors:** Luyu Xie, Jaime P. Almandoz, M. Sunil Mathew, Jeffrey N. Schellinger, Olivia Kapera, Sophia Ngenge, Elisa Morales Marroquin, Carrie McAdams, Sachin Kukreja, Benjamin Schneider, Sarah E. Messiah

**Affiliations:** 1 School of Public Health, University of Texas Health Science Center, Dallas; 2 Center for Pediatric Population Health, UTHealth School of Public Health, Dallas, Texas; 3 Division of Endocrinology, Department of Internal Medicine, University of Texas Southwestern Medical Center, Dallas; 4 School of Public Health, University of Texas Health Science Center, Austin; 5 Department of Psychiatry, University of Texas Southwestern Medical Center, Dallas; 6 Minimally Invasive Surgical Associates, Dallas, Texas; 7 Department of Surgery, University of Texas Southwestern Medical Center, Dallas

## Abstract

**Importance:**

Metabolic and bariatric surgery (MBS) is an effective and safe treatment for obesity and its comorbidities, but less than 50% of those who are eligible and referred for MBS complete the procedure. The patient-physician relationship could be a decisive factor in the decision to complete MBS; however, this relationship has not been explored, particularly among racially and ethnically diverse populations.

**Objective:**

To examine the association between patient-reported satisfaction with their patient-physician relationship and MBS completion by self-reported racial and ethnic group.

**Design, Setting, and Participants:**

This prospective cohort study included 408 patients who were referred to a bariatric surgeon or obesity medicine program between July 24, 2019, and May 19, 2022.

**Exposure:**

Patient satisfaction with their physician was measured by 7 dimensions (general satisfaction, technical quality, interpersonal manner, communication, financial aspects, time spent with physician, and accessibility and convenience) using the Patient Satisfaction Questionnaire Short Form.

**Main Outcomes and Measures:**

Completion status for MBS (yes or no).

**Results:**

A total of 408 patients (mean [SD] age, 47.3 [11.6] years; among 366 with data available, 317 [86.6%] women and 49 [13.4%] men) were included in the study. Of 363 patients with data available on race and ethnicity, 66 were Hispanic/Latinx (18.2%), 136 (37.5%) were non-Hispanic Black, 146 (40.2%) were non-Hispanic White, and 15 (4.1%) were other race or ethnicity. A total of 124 patients (30.4%) completed MBS. Overall, the mean (SD) patient satisfaction score was significantly greater in MBS completers vs noncompleters (3.86 [0.56] vs 3.61 [0.64]; *P* < .001). Multivariable logistic regression analysis showed technical quality was the most significant factor for MBS completion (adjusted odds ratio [aOR], 1.99 [95% CI, 1.24-3.19]), followed by communication (aOR, 1.78 [95% CI, 1.16-2.72]) and accessibility and convenience (aOR, 1.61 [95% CI, 1.03-2.53]). The interaction between racial and ethnic groups and patient satisfaction was not significant (eg, mean [SD] score for Hispanic/Latinx completers, 3.95 [0.55] vs 3.77 [0.60] for non-Hispanic White completers; *P* = .46 for interaction).

**Conclusions and Relevance:**

These findings suggest that there is an association between patient satisfaction with the patient-physician relationship and the decision to complete MBS regardless of race and ethnicity. These findings have important implications for strategies to improve the proportion of qualified patients who complete MBS to achieve improved health outcomes.

## Key Points

**Question:**

Is there an association between patient satisfaction with their patient-physician relationship and the decision to complete metabolic and bariatric surgery (MBS), and is race and ethnicity an independent factor in this association?

**Findings:**

In this cohort study that included 408 adults, the mean patient satisfaction score was significantly greater among those who completed MBS vs those who did not regardless of racial and ethnic identity.

**Meaning:**

These findings suggest that patient satisfaction with their patient-physician relationship was associated with completion of MBS among all racial and ethnic groups.

## Teaser

This cohort study examines the association between the patient-physician relationship and the decision to complete metabolic and bariatric surgery (MBS) and assesses whether race and ethnicity is an independent factor associated with MBS completion.

## Introduction

At present, 41.9% of US adults have obesity (body mass index [BMI; calculated as weight in kilograms divided by height in meters squared] ≥30.0),^1^ and future projections show half of adults will have obesity by 2030.^2^ Even more alarming, over the past 20 years, the number of US adults with class III obesity^3^ (also known as severe obesity, defined as BMI ≥40.0) has increased by 50%.^4^ Moreover, racial and ethnic group disparities remain entrenched in the obesity epidemic. In 2020, 45.6% of Hispanic/Latinx adults and 49.9% of non-Hispanic Black adults in the US had obesity, and 7.4% and 14.0% had class III obesity, respectively.^1,5^ Severe obesity is a risk factor for many chronic health conditions, including cardiometabolic diseases and certain types of cancers, and was associated with nearly $200 billion in health care costs in 2019.^1^

Metabolic and bariatric surgery (MBS) is an effective and safe treatment for obesity.^6,7,8^ Metabolic and bariatric surgery can also improve obesity-related comorbidities, including type 2 diabetes, dyslipidemia, and hypertension.^6,7,8^ However, MBS is an underused tool for treating obesity, as only about one-half of individuals who are eligible and referred for MBS complete it. A recent population-based study^9^ showed the MBS utilization rate was only 5.56 per 1000 eligible adults in the US. Most importantly, racial and ethnic group disparities are present in MBS utilization rates. Specifically, it was reported that 57.8% of MBS completers were non-Hispanic White, whereas only 17.3% were non-Hispanic Black and 9.4% were Hispanic.^9^ In terms of disparities in post-MBS outcomes, studies have shown that non-Hispanic Black patients have a higher incidence of 30-day post-MBS complications compared with their non-Hispanic White counterparts and lower long-term, sustained weight loss compared with non-Hispanic White and Hispanic/Latinx patients.^10,11,12^ However, other studies have shown that MBS reduces cardiometabolic disease risk in Hispanic/Latinx patients^13^ and reduces the risk of type 2 diabetes and prediabetes among multiethnic adults.^14^ Qualitative studies among primarily non-Hispanic Black and Hispanic/Latinx MBS completers^15^ have shown that comorbidity resolution, including mobility improvements, are primary reasons for MBS completion.

However, the personal rationale for the low utilization rate of MBS is largely unknown, and even less is known about why these rates vary so much by ethnicity. Lack of insurance coverage for MBS, perceived invasiveness and irreversibility, concerns about surgical complications,^16^ nutritional deficiencies, and the lack of physicians who speak the patient’s native language, primarily Spanish, are reasons cited to date for this attrition.^17,18,19,20,21,22,23^ There is also a growing interest in the role the patient-physician relationship plays in treating obesity, because patients have been shown to be generally highly receptive to health advice from their physicians in other domains (eg, smoking).^24,25,26^ Miscommunication with the primary care physician has been cited as a barrier to MBS completion abroad,^27,28,29^ but this has not been examined across racially and ethnically diverse patient groups in the US, which has prompted the call for more research in this area.

To fill this important gap in the literature, we examined the association between the patient-physician relationship and the decision to complete MBS among a racially and ethnically diverse group of patients. We hypothesized that higher patient satisfaction with their patient-physician relationship would increase the likelihood of completing MBS.

## Methods

This study followed the Strengthening the Reporting of Observational Studies in Epidemiology (STROBE) reporting guideline and was approved by the University of Texas Health Science Center, Houston, Committee for Protection of Human Subjects. All participants provided written informed consent.

### Study Design and Population

There are 2 phases of this ongoing prospective cohort study (the Bariatric Health Study): (1) to identify the socioecological characteristics associated with patient-directed completion or noncompletion of MBS and (2) to determine the short-term (6-month), midterm (12-month), and long-term (24-month) changes in these characteristics and how they are associated with weight loss and comorbidity resolution in an ethnically diverse patient population in North Texas. Herein, we report the findings from the phase 1 study that focuses on patient satisfaction with their physicians.

Participants were recruited from an MBS clinic at an academic center, an MBS community clinic, and an obesity medicine clinic at an academic center that manages patients before and after MBS, located in the Dallas–Fort Worth metroplex. To be eligible for the study, participants were required to (1) meet the National Institutes of Health criteria to qualify for MBS^30^ and (2) consent to participate in the study. Patients were excluded if they did not meet inclusion criteria or were younger than 18 years. Patients were referred for MBS between July 24, 2019, and May 19, 2022.

### Study Procedures and Recruitment Strategies

Most patients in the Bariatric Health Study were referred for weight management, primarily by their primary care physicians, to an MBS practice or an obesity care clinic. Eligible patients completed a mandatory MBS educational seminar before scheduling surgery. Clinic staff recruited patients to the Bariatric Health Study during the seminar. Specifically, at each seminar, the MBS team, including surgeons, dietitians, nurse practitioners, physician assistants, registered nurses, and intake coordinators, discussed the following topics: (1) what obesity is and how it impacts overall health; (2) who is eligible for MBS; (3) lifestyle changes before and after MBS; (4) the types of MBS procedures they offer, the benefits and risks of each, and their expected results; and (5) payment options and insurance coverage questions. Other recruitment strategies included flyers and brochures posted at various obesity clinics and on a social media page created by research study staff to provide study information via an institutional review board–approved study flyer and to respond to any questions from interested participants.

Eligible patients first participated in a 15- to 20-minute telephone or video interview in which trained study team members administered the verbal portion of questionnaires. On completion of the interview, participants were directed to complete the self-administered online questionnaire, which was delivered via email through REDCap. Participants were compensated with an emailed gift card ($50 for the baseline or presurgery interview) for their participation after completion of the questionnaires.

### Measurement and Assessment

#### Exposures

Patient satisfaction was the primary exposure assessed by the 18-item Patient Satisfaction Questionnaire Short Form (PSQ-18).^31^ The PSQ-18 is validated to measure patient satisfaction using 18 items in 7 different dimensions, including general satisfaction (items 3 and 17), technical quality (items 2, 4, 6, and 14), interpersonal manner (items 10 and 11), communication (items 1 and 13), financial aspects (items 5 and 7), time spent with the physician (items 12 and 15), and accessibility and convenience (items 8, 9, 16, and 18). Each dimension includes a mixture of positive and negative questions, and each question contains a 5-point Likert scale (strongly agree, agree, uncertain, disagree, and strongly disagree). After applying the specific scoring methods (eTable in Supplement 1), patient satisfaction was converted into a mean score ranging from 1 to 5, in which a higher score indicates greater satisfaction. The PSQ-18 was administered during the self-administrated online interview.

#### Outcomes

Completion of MBS (the primary outcome) was self-reported by patients, collected by trained research staff during the interview, and verified by the MBS and obesity medicine clinical staff. It was categorized into a binary variable of completion vs no completion.

Covariates included the following sociodemographic factors: age (continuous variable), sex (man or woman), self-reported race and ethnicity (Hispanic/Latinx, non-Hispanic Black, non-Hispanic White, and other race or ethnicity [including American Indian or Alaska Native, Asian, Native Hawaiian or other Pacific Islander, and >1 race or ethnicity]), payer status (government insurance, private insurance, or no insurance), and BMI [<30.0, 30.0-34.9, 35.0-39.9, 40.0-49.9, or ≥50.0]). These covariates were chosen because previous studies have suggested they are associated with behaviors surrounding MBS utilization.^19,20,21,22,23,24,25,26,27,28,29^

### Statistical Analysis

For descriptive analysis, categorical variables are presented as frequencies (percentages), and continuous variables are presented as mean (SD). The patient satisfaction score was treated as a continuous variable and is presented as mean (SD) throughout the analysis. To compare patient satisfaction scores by MBS completion status, a 2-sample *t* test with equal or unequal variance was used. To assess patient satisfaction scores by race and ethnicity, a 1-way analysis of variance with Bonferroni correction was performed to calculate *P* values.

Univariable logistic regression models were built to examine the association between MBS completion (dependent variable) and patient satisfaction or race and ethnicity (independent variables). Multivariable models also adjusted for age, sex, BMI, and insurance status and were used to compute adjusted odds ratios (aORs). We also performed interaction analysis via a multivariable logistic regression model, adding the interaction term of race and ethnicity × mean PSQ-18 score to examine whether there was a modifying association with race and ethnicity. All analyses were performed using SAS, version 9.4 (SAS Institute Inc). Two-sided *P* < .05 was considered statistically significant.

This study aimed to recruit at least 400 participants to provide approximately 80% power at the level of α = .01 as long as the effect accounted for 3% of the total variance relative to the model specific error term (ie, *R*^2^ = 0.03).^32^ Because we were able to recruit 408 patients in the phase 1 study, a post hoc power analysis was performed and found our study had greater than 99.9% statistical power to detect differences between completers and noncompleters at the level of α = .05.

## Results

A total of 408 patients (mean [SD] age, 47.3 [11.6] years; among 366 with data available, 317 [86.6%] women and 49 [13.4%] men) were included in the final analytical sample. Among the 363 patients with data available, 66 (18.2%) were Hispanic/Latinx; 136 (37.5%), non-Hispanic Black; 146 (40.2%), non-Hispanic White; and 15 (4.1%), other. Most patients (333 of 340 [97.9%]) had a BMI of 30.0 or greater and were covered by private insurance (163 of 233 [70.0%]). Seven patients (2.1%) were referred from their primary care physician to an MBS program but at the time of their visit were ineligible for surgery owing to a BMI of less than 30.0 and thus were not scheduled to complete MBS. Instead, they were referred to the weight management program and thus were included in the noncompleter group. In our sample, 124 patients (30.4%) have completed MBS to date. There were no differences in patients’ characteristics, including age, sex, race and ethnicity, BMI, and educational attainment by MBS completion status. However, significantly more noncompleters had no insurance compared with completers (12 of 154 [7.8%] vs 1 of 79 [1.3%]; *P* = .04) (Table 1).

**Table 1.** Participant Characteristics by MBS Completion Status^a^

Characteristic	MBS group			*P* value^b^
	All (N = 408)	Completers (n = 124)	Noncompleters (n = 284)	
Age, mean (SD), y	47.3 (11.6)	46.0 (10.8)	47.9 (12.1)	.13
Sex				
Women	317/366 (86.6)	109/124 (87.9)	208/242 (86.0)	.60
Men	49/366 (13.4)	15/124 (12.1)	34/242 (14.0)	
Race and ethnicity				
Hispanic/Latinx	66/363 (18.2)	18/124 (14.5)	48/239 (20.1)	.62
Non-Hispanic Black	136/363 (37.5)	48/124 (38.7)	88/239 (36.8)	
Non-Hispanic White	146/363 (40.2)	53/124 (42.7)	93/239 (38.9)	
Other^c^	15/363 (4.1)	5/124 (4.0)	10/239 (4.2)	
BMI				
<30.0	7/340 (2.1)	0/122	7/218 (3.2)	.07
30.0-34.9	19/340 (5.6)	3/122 (2.5)	16/218 (7.3)	
35.0-39.9	78/340 (22.9)	26/122 (21.3)	52/218 (23.9)	
40.0-49.9	145/340 (42.6)	58/122 (47.5)	87/218 (39.9)	
≥50.0	91/340 (26.8)	35/122 (28.7)	56/218 (25.7)	
Insurance status				
Government	57/233 (24.5)	25/79 (31.6)	32/154 (20.8)	.04
Private	163/233 (70.0)	53/79 (67.1)	110/154 (71.4)	
None	13/233 (5.6)	1/79 (1.3)	12/154 (7.8)	
Educational attainment				
High school or below	94/334 (28.1)	31/123 (25.2)	63/211 (29.9)	.44
College	148/334 (44.3)	60/123 (48.8)	88/211 (41.7)	
Graduate or professional degree	92/334 (27.5)	32/123 (26.0)	60/211 (28.4)	

Abbreviations: BMI, body mass index (calculated as weight in kilograms divided by height in meters squared); MBS, metabolic and bariatric surgery.

^a^ Unless otherwise indicated, data are expressed as No./total No. (%) of patients with data available. Owing to missing data in categories, denominators do not always total numbers in column headings. Percentages have been rounded and may not total 100.

^b^ Calculated using χ^2^ analysis.

^c^ Includes American Indian or Alaska Native, Asian, Native Hawaiian or other Pacific Islander, and more than 1 race or ethnicity.

Table 2 compares patient satisfaction between MBS completers and noncompleters. Specifically, results showed statistically significant differences between the 2 groups in all PSQ-18 subdomains, including a higher general satisfaction (3.95 [0.82] vs 3.66 [0.95]; *P* = .005), technical quality (3.97 [0.69] vs 3.69 [0.73]; *P* < .001), interpersonal manner (4.07 [0.65] vs 3.95 [0.79]; *P* = .004), communication (4.00 [0.75] vs 3.60 [0.81]; *P* = .002), financial aspects (3.42 [1.03] vs 3.14 [1.09]; *P* = .02), and accessibility and convenience (3.80 [0.68] vs 3.56 [0.71]; *P* = .003), with the exception of time spent with physician (3.67 [0.88] vs 3.53 [0.93]; *P* = .16). Overall, the mean satisfaction score of MBS completers was significantly greater than that of the noncompleters (3.86 [0.56] vs 3.61 [0.64]; *P* < .001), with an overall satisfaction score of 3.70 (0.62).

**Table 2.** Patient Satisfaction Assessment by Bariatric Surgery Completion Status Using the PSQ-18 Subscales and Constituent Items^a^

PSQ-18 item	MBS group score, mean (SD)			*P* value^b^
	All (N = 408)	Completers (n = 124)	Noncompleters (n = 284)	
General satisfaction	3.77 (0.91)	3.95 (0.82)	3.66 (0.95)	.005
3. The medical care I have been receiving is just about perfect.	4.00 (0.99)	4.18 (0.91)	3.89 (1.02)	.01
17. I am dissatisfied with some things about medical care I receive.	3.54 (1.10)	3.72 (1.03)	3.43 (1.12)	.02
Technical quality	3.79 (0.72)	3.97 (0.69)	3.69 (0.73)	<.001
2. I think my physician’s office has everything needed to provide complete care.	4.21 (0.86)	4.37 (0.76)	4.12 (0.91)	.007
4. Sometimes physicians make me wonder if their diagnosis is correct.	3.16 (1.18)	3.35 (1.17)	3.05 (1.17)	.03
6. When I go for medical care, they are careful to check everything when treating and examining me.	3.92 (0.98)	4.09 (0.83)	3.82 (1.04)	.01
14. I have some doubts about the ability of physicians who treat me.	3.86 (0.98)	4.06 (0.88)	3.75 (1.02)	.006
Interpersonal manner	4.04 (0.75)	4.07 (0.65)	3.95 (0.79)	.004
10. Physicians act too business like and impersonal toward me.	3.78 (0.99)	3.96 (0.84)	3.66 (1.05)	.003
11. My physicians treat me in a very friendly and courteous manner.	4.30 (0.81)	4.40 (0.72)	4.25 (0.86)	.09
Communication	3.82 (0.80)	4.00 (0.75)	3.60 (0.81)	.002
1. Physicians are good about explaining the reason for medical tests.	4.15 (0.89)	4.28 (0.83)	4.07 (0.92)	.04
13. Physicians sometimes ignore what I tell them.	3.48 (1.08)	3.70 (1.05)	3.35 (1.08)	.004
Financial aspects	3.24 (1.07)	3.42 (1.03)	3.14 (1.09)	.02
5. I feel confident that I can get the medical care I need without being set back financially.	3.42 (1.26)	3.63 (1.16)	3.29 (1.30)	.02
7. I have to pay for more of my medical care than I can afford.	3.08 (1.29)	3.21 (1.21)	3.0 (1.33)	.15
Time spent with physician	3.57 (0.91)	3.67 (0.88)	3.53 (0.93)	.16
12. Those who provide my medical care sometimes hurry too much when they treat me.	3.44 (1.12)	3.59 (1.13)	3.36 (1.11)	.08
15. Physicians usually spend plenty of time with me.	3.71 (1.07)	3.76 (1.07)	3.69 (1.05)	.58
Accessibility and convenience	3.65 (0.70)	3.80 (0.68)	3.56 (0.71)	.003
8. I have easy access to the medical specialists I need.	4.02 (1.05)	4.16 (0.88)	3.93 (1.12)	.04
9. Where I get medical care, people have to wait too long for emergency treatment.	3.33 (1.11)	3.50 (1.01)	3.23 (1.16)	.03
16. I find it too hard to get an appointment for medical care right away.	3.33 (1.16)	3.47 (1.18)	3.24 (1.14)	.08
18. I am able to get medical care whenever I need it.	3.95 (0.89)	4.07 (0.87)	3.88 (0.90)	.07
Overall satisfaction score	3.70 (0.62)	3.86 (0.56)	3.61 (0.64)	<.001

Abbreviations: MBS, metabolic and bariatric surgery; PSQ-18, 18-item Patient Satisfaction Questionnaire Short Form.

^a^ Scores range from 1 (strongly agree [least satisfied]) to 5 (strongly disagree [most satisfied]); items 1, 2, 3, 5, 6, 8, 11, 15, and 18 were inversely scored (eTable in Supplement 1).

^b^ Calculated using a 2-sample *t* test with equal or unequal variance.

In the aggregate study sample, the subdomain with the greatest patient satisfaction was interpersonal manner (PSQ-18 score, 4.04 [0.75]), followed by communication (PSQ-18 score, 3.82 [0.80]), technical quality (PSQ-18 score, 3.79 [0.72]), general satisfaction (PSQ-18 score, 3.77 [0.91]), accessibility and convenience (PSQ-18 score, 3.65 [0.70]), and time spent with physician (PSQ-18 score, 3.57 [0.91]). The subdomain with the least patient satisfaction was financial aspects (PSQ-18 score, 3.24 [1.07]). There were some variations in PSQ-18 score for each question within a subdomain. For example, in terms of technical quality, the patient-reported satisfaction score was only 3.16 (1.18) for the second question, “Sometimes physicians make me wonder if their diagnosis is correct,” but it was 4.21 (0.86) for the first question, “I think my physician’s office has everything needed to provide complete care” (Table 2).

Table 3 demonstrates the pairwise comparison of patient satisfaction among MBS completers by race and ethnicity. No ethnic differences were found in the overall mean satisfaction scores (Hispanic/Latinx patients, 3.95 [0.55]; non-Hispanic Black patients, 3.94 [0.48]; non-Hispanic White patients, 3.77 [0.60]; and other patients, 3.74 [0.72) or subdomains (all *P* > .05). Specifically, the domain with the least satisfaction was interpersonal manner for all ethnic groups: (4.28 [0.54] for Hispanic/Latinx patients; 4.28 [0.68] for non-Hispanic Black patients; 4.09 [0.62] for non-Hispanic White patients; and 4.00 [1.00] for other patients). Financial aspects was found to be the subdomain with the least satisfaction among all ethnic groups (3.33 [1.10] for Hispanic/Latinx patients, 3.45 [0.97] for non-Hispanic Black patients, 3.46 [1.09] for non-Hispanic White patients, and 3.10 [0.55] for other patients).

**Table 3.** Patient Satisfaction Assessment by Race and Ethnicity Using the PSQ-18 Subscales and Constituent Items Among MBS Completers^a^^,^^b^

PSQ-18 item	Score by racial and ethnic group, mean (SD)				
	Total (N = 124)	Hispanic/Latinx (n = 18)	Non-Hispanic Black (n = 48)	Non-Hispanic White (n = 53)	Other (n = 5)^c^
General satisfaction	3.95 (0.82)	4.14 (0.56)	4.09 (0.79)	3.77 (0.89)	3.90 (0.96)
3. The medical care I have been receiving is just about perfect.	4.18 (0.91)	4.39 (0.51)	4.26 (0.82)	4.04 (1.07)	4.20 (0.84)
17. I am dissatisfied with some things about medical care I receive.	3.72 (1.03)	3.89 (1.02)	3.91 (0.97)	3.51 (1.07)	3.60 (1.14)
Technical quality	3.97 (0.69)	4.08 (0.58)	4.03 (0.62)	3.88 (0.75)	3.85 (0.94)
2. I think my physician’s office has everything needed to provide complete care.	4.37 (0.76)	4.44 (0.62)	4.51 (0.62)	4.23 (0.89)	4.40 (0.89)
4. Sometimes physicians make me wonder if their diagnosis is correct.	3.35 (1.17)	3.33 (1.14)	3.23 (1.25)	3.42 (1.12)	3.80 (1.30)
6. When I go for medical care, they are careful to check everything when treating and examining me.	4.09 (0.83)	4.33 (0.59)	4.15 (0.83)	3.96 (0.85)	4.00 (1.22)
14. I have some doubts about the ability of physicians who treat me.	4.06 (0.88)	4.22 (0.88)	4.23 (0.73)	3.92 (0.85)	3.20 (1.79)
Interpersonal manner	4.07 (0.65)	4.28 (0.54)	4.28 (0.68)	4.09 (0.62)	4.00 (1.00)
10. Physicians act too business like and impersonal toward me.	3.96 (0.84)	4.00 (0.91)	4.06 (0.87)	3.92 (0.78)	3.60 (1.14)
11. My physicians treat me in a very friendly and courteous manner.	4.40 (0.72)	4.56 (0.51)	4.49 (0.72)	4.26 (0.76)	4.40 (0.89)
Communication	4.00 (0.75)	4.03 (0.63)	4.19 (0.59)	3.80 (0.87)	4.10 (0.65)
1. Physicians are good about explaining the reason for medical tests.	4.28 (0.83)	4.50 (0.51)	4.45 (0.72)	4.07 (0.98)	4.20 (0.45)
13. Physicians sometimes ignore what I tell them.	3.70 (1.05)	3.56 (1.10)	3.93 (0.90)	3.53 (1.12)	4.00 (1.22)
Financial aspects	3.42 (1.03)	3.33 (1.10)	3.45 (0.97)	3.46 (1.09)	3.10 (0.55)
5. I feel confident that I can get the medical care I need without being set back financially.	3.63 (1.16)	3.67 (1.03)	3.74 (1.03)	3.55 (1.32)	3.40 (1.14)
7. I have to pay for more of my medical care than I can afford.	3.21 (1.21)	3.0 (1.41)	3.15 (1.23)	3.38 (1.15)	2.80 (0.84)
Time spent with physician	3.67 (0.88)	3.64 (0.92)	3.69 (0.83)	3.68 (0.95)	3.50 (0.71)
12. Those who provide my medical care sometimes hurry too much when they treat me.	3.59 (1.13)	3.39 (1.19)	3.77 (1.05)	3.47 (1.17)	3.80 (1.30)
15. Physicians usually spend plenty of time with me.	3.76 (1.07)	3.89 (0.96)	3.62 (1.23)	3.89 (0.93)	3.20 (1.30)
Accessibility and convenience	3.80 (0.68)	3.97 (0.64)	3.85 (0.68)	3.70 (0.67)	3.70 (0.84)
8. I have easy access to the medical specialists I need.	4.16 (0.88)	4.39 (0.61)	4.23 (0.89)	4.06 (0.91)	3.80 (1.30)
9. Where I get medical care, people have to wait too long for emergency treatment.	3.50 (1.01)	3.56 (1.10)	3.60 (1.10)	3.38 (0.92)	3.80 (0.84)
16. I find it too hard to get an appointment for medical care right away.	3.47 (1.18)	3.72 (0.96)	3.40 (1.39)	3.45 (1.07)	3.40 (1.14)
18. I am able to get medical care whenever I need it.	4.07 (0.87)	4.22 (0.55)	4.17 (0.88)	3.94 (0.95)	3.80 (0.84)
Overall satisfaction score	3.86 (0.56)	3.95 (0.55)	3.94 (0.48)	3.77 (0.60)	3.74 (0.72)

Abbreviations: MBS, metabolic and bariatric surgery; PSQ-18, 18-item Patient Satisfaction Questionnaire Short Form.

^a^ Scores range from 1 (strongly agree [least satisfied]) to 5 (strongly disagree [most satisfied]); items 1, 2, 3, 5, 6, 8, 11, 15, and 18 were inversely scored (eTable in Supplement 1).

^b^ Using pairwise analysis of variance with Bonferroni correction, all *P* > .05.

^c^ Includes American Indian or Alaska Native, Asian, Native Hawaiian or other Pacific Islander, and more than 1 race or ethnicity.

Univariable and multivariable logistic regression models showed that higher overall mean PSQ-18 scores (ie, better patient satisfaction) were associated with higher odds of pursuing MBS (crude OR, 2.00 [95% CI, 1.36-2.94]; *P* < .001; and aOR, 1.93 [95% CI, 1.13-3.29]; *P* = .02). The multivariable model suggested that with a 1-point increase in the satisfaction score of technical quality, the odds of completing MBS doubled (aOR, 1.99 [95% CI, 1.24-3.19]; *P* = .004), which was the most significant factor found. Additionally, communication (aOR, 1.78 [95% CI, 1.16-2.72]) and accessibility and convenience (aOR, 1.61 [95% CI, 1.03-2.53]) were significant factors. Race and ethnicity was not an independent factor associated with MBS completion (aOR for Hispanic/Latinx patients, 0.72 [95% CI, 0.30-1.72]; aOR for non-Hispanic Black patients, 0.99 [95% CI, 0.52-1.91]; aOR for other race and ethnicity, 0.93 [95% CI, 0.15-5.65]; all *P* > .05) (Table 4). We performed an interaction analysis to further assess race and ethnicity as a potential moderating factor, as shown in the Figure. Among completers, mean (SD) PSQ-18 scores of Hispanic/Latinix patients were 3.95 (0.55); non-Hispanic Black patients, 3.94 (0.48); non-Hispanic White patients, 3.77 (0.60); and patients of other race or ethnic group, 3.74 (0.72). Among noncompleters, mean (SD) PSQ-18 scores for Hispanic/Latinx patients were 3.49 (0.71); non-Hispanic Black patients, 3.68 (0.60); non-Hispanic White patients, 3.65 (0.61); and patients of other race of ethnicity, 3.17 (0.66). The overall interaction between racial and ethnic groups and patient satisfaction was insignificant (*P* = .46 for interaction).

**Table 4.** Association of Patient Satisfaction and Decision on Metabolic and Bariatric Surgery With Race and Ethnicity

Independent variables	Univariable model^a^		Multivariable model^b^	
	OR (95% CI)	*P* value	aOR (95% CI)	*P* value
PSQ-18 items				
Overall	2.00 (1.36-2.94)	<.001	1.93 (1.13-3.29)	.02
General satisfaction	1.45 (1.12-1.88)	.006	1.38 (0.96-1.97)	.08
Technical quality	1.75 (1.26-2.43)	<.001	1.99 (1.24-3.19)	.004
Interpersonal manner	1.56 (1.13-2.16)	.007	1.33 (0.86-2.05)	.20
Communication	1.60 (1.18-2.16)	.002	1.78 (1.16-2.72)	.008
Financial aspects	1.29 (1.04-1.59)	.02	1.14 (0.85-1.53)	.38
Time spent with physician	1.19 (0.93-1.53)	.16	1.14 (0.82-1.59)	.43
Accessibility and convenience	1.63 (1.17-2.26)	.004	1.61 (1.03-2.53)	.04
Race and ethnicity				
Hispanic/Latinx	0.66 (0.35-1.25)	.29	0.72 (0.30-1.72)	.46
Non-Hispanic Black	0.96 (0.59-1.56)	.62	0.99 (0.52-1.91)	.99
Non-Hispanic White	1 [Reference]	NA	1 [Reference]	NA
Other^c^	0.88 (0.29-2.70)	.97	0.93 (0.15-5.65)	.94

Abbreviations: aOR, adjusted odds ratio; NA, not applicable; OR, odds ratio; PSQ-18, 18-item Patient Satisfaction Questionnaire Short Form.

^a^ Univariable logistic regression models using surgery completion (yes or no) as the dependent variable controlling for each independent variable (race and ethnicity or PSQ-18 items).

^b^ Multivariable logistic regression models using surgery completion (yes or no) as the dependent variable controlling for independent variables (race and ethnicity [non-Hispanic White as the reference group] or PSQ-18 items [continuous scale]) and age, sex, body mass index, and insurance status.

^c^ Includes American Indian or Alaska Native, Asian, Native Hawaiian or other Pacific Islander, and more than 1 race or ethnicity.

**Figure.**
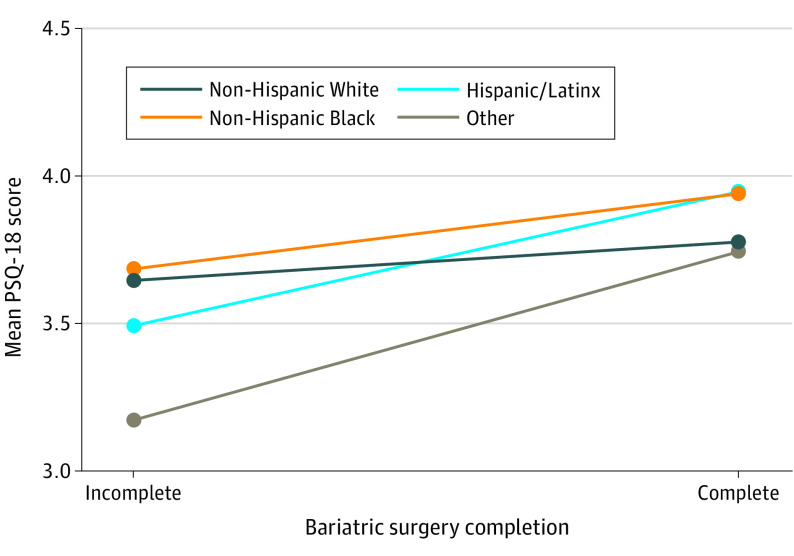
Interaction Between Race and Ethnicity and Patient Satisfaction on the Decision to Pursue Metabolic and Bariatric Surgery Other race and ethnicity includes American Indian or Alaska Native, Asian, Native Hawaiian or other Pacific Islander, and more than 1 race or ethnicity. *P* = .46 for interaction overall; *P* = .29 for interaction, non-Hispanic Black × non-Hispanic White patients; *P* = .20 for interaction, Hispanic/Latinx × non-Hispanic White patients; and *P* = .32 for interaction, other × non-Hispanic White patients. PSQ-18 indicates 18-item Patient Satisfaction Questionnaire Short Form.

## Discussion

This cohort study found patient satisfaction, as measured by the PSQ-18, to be a significant factor of MBS completion regardless of race or ethnicity. This finding aligns with those of previous literature that the patient-physician relationship is associated with quality and efficiency of surgical care,^33,34^ greater adherence, compliance, persistence to treatment, retention, and use of health care services in different settings.^35,36,37,38^ However, few studies have examined the association between patient satisfaction and the decision to pursue and complete MBS. Given that members of racial and ethnic minority groups, such as non-Hispanic Black individuals, disproportionally experience chronic obesity and related conditions,^6,7,8^ racial and ethnic disparities in MBS completion rates cannot be neglected. Our results suggest that enhancing patient-physician relationships for all racial and ethnic groups may help improve the MBS utilization rate.

In this study, we found that one of the most significant factors for MBS completion was technical quality, which was assessed by the following 4 questions: (1) “I think my physician’s office has everything needed to provide complete care”; (2) “Sometimes physicians make me wonder if their diagnosis is correct”; (3) “When I go for medical care, they are careful to check everything when treating and examining me”; and (4) “I have some doubts about the ability of physicians who treat me.” Despite the patient satisfaction score, with technical quality being slightly greater than the overall patient satisfaction score (3.79 [0.72] and 3.70 [0.62], respectively), the score varied for each item. For example, the patient-reported satisfaction score was only 3.16 (1.18) for the second question above, but it was 4.21 (0.86) for the first question. This finding is consistent with a recent American Board of Internal Medicine survey suggesting that 17% of US patients distrust health care systems in general.^39^ This indicates that more effort may be needed to build trust with patients because the ability of a physician to gain patient trust has been shown to be the foundation of a good patient-physician relationship.^40^

Studies have shown that racial discrimination exists in health care settings.^41,42^ Non-Hispanic Black and Hispanic/Latinx patients tend to have a lower level of trust in the health care system than non-Hispanic White patients.^41,42^ Non-Hispanic Black patients are more likely to have concerns about privacy or the potential for harmful experimentation during treatment.^41^ In addition, men usually have more distrust than women.^42^ Hence, trust in the health care system differs by race and ethnicity and sex and can hinder treatment decisions, adherence, and compliance.

Satisfaction with communication and accessibility and convenience can also be targeted as a strategy to improve the number of eligible patients completing MBS. Excellent communication skills are one of the most critical components in enabling the allocation of trust from patient to surgeon.^40^ Enhancing communication will not only improve patient satisfaction but also improve health care outcomes.^43^ On the other hand, being unable to access health care and lack of convenience is associated with poor adherence, unsatisfied patient experiences, and lower levels of health care services used.^44,45^ Our study was conducted mostly during the COVID-19 pandemic, and masking and social distancing also had negative impacts on establishing the patient-physician relationship because nonverbal communication via facial expressions is an essential component for building and maintaining a good relationship between patients and physicians.^46^ Wearing masks and social distancing will decrease patients’ perceptions of physician empathy.^47^ Additionally, 30% to 50% of US residents had reduced health care access during the pandemic,^48^ which may lead to distant relationships with their physicians.

Several strategies can be used to improve the patient-physician relationship, particularly in the context of the decision to pursue MBS. Shared decision-making is an effective method to facilitate patient engagement and health care outcomes. This strategy has 3 major steps: introducing choice, describing options, and helping patients explore preferences and make decisions.^49^ When physicians spend adequate time with patients when making a decision, the patients are more likely to have better satisfaction and undergo MBS.^49,50^ Another strategy is attachment theory, which holds that “cognitive schemas based on earlier repeated experiences with caregivers influence how individuals perceive and act within interpersonal relationships.”^51^ Specifically, according to attachment theory, patients develop attachment to physicians if they feel secure with them and that the provided care is genuine. One study^52^ showed that attachment between patients and physicians was positively associated with continuity of health care visits, use of health care services, and symptom perceptions.

Although we did not find any differences in terms of race and ethnicity with regard to MBS completion, the aforementioned strategies can be tailored to different racial and ethnic groups. Previous studies have suggested that racial concordance between physicians and patients may improve health care outcomes.^53,54^ The patient-physician relationship is enhanced when patients perceive similarities with their physicians. Despite racial concordance being one of the most significant factors for perceiving personal similarity, to improve health care outcomes among a diverse patient population, it may be necessary to train racially discordant patient-physician dyads on how to improve engagement, the quality of communication, and patient centeredness and to build trust.^54^

### Limitations

This study has limitations. First, we used a sample of convenience, and all participants were recruited from a single large geographic region; therefore, our results may not be generalizable to other geographic locations in the US or abroad. Due to data limitations, we did not collect comorbidity information, resulting in residual confounding, because health factors are important for MBS completion.^50^ Additionally, although the MBS completion status was self-reported, which may be prone to recall or reporting bias, our research staff verified patient health records to verify responses. Despite these limitations, our study is, to our knowledge, the first in the literature to examine the role of the patient-physician relationship in the decision to complete MBS among a racially and ethnically diverse sample using a standardized measurement tool.

## Conclusions

The findings of this prospective cohort study suggest that patient satisfaction with their patient-physican relationship was associated with MBS completion regardless of racial and ethnic group. Specifically, improving patient satisfaction by building trust with physicians’ technical skills, enhancing communication, and improving accessibility to health care visits may increase MBS utilization to treat obesity and its complications among all racial and ethnic groups.

## Notes

Supplement 1. **eTable.** PSQ-18 Scoring Items

Supplement 2.
Data Sharing Statement
