# Supplementary material for: Association Between Patient Satisfaction With Their Patient-Physician Relationship and Completion of Bariatric Surgery by Race and Ethnicity Among US Adults
Source: JAMA Netw Open. 2022 Dec 19;5(12):e2247431. doi: 10.1001/jamanetworkopen.2022.47431 (PMC9856898; doi:10.1001/jamanetworkopen.2022.47431)
Supplement: Supplement 1. — eTable. PSQ-18 Scoring Items [file jamanetwopen-e2247431-s001.pdf]

Supplementary Online Content

Xie L, Almandoz JP, Mathew MS, et al. Association between patient satisfaction with their patient-physician relationship and completion of bariatric surgery by race and ethnicity among US adults. *JAMA Netw Open*. 2022;5(12):e2247431. doi:10.1001/jamanetworkopen.2022.47431

eTable. PSQ-18 Scoring Items

This supplementary material has been provided by the authors to give readers additional information about their work.

© 2022 Xie L et al. *JAMA Network Open*.

eTable. PSQ-18 Scoring Items

Item numbers	Original value	Scored Value
1, 2, 3, 5, 6, 8, 11, 15, 18	1 = strongly agree	5
	2 = agree	4
	3 = uncertain	3
	4 = disagree	2
	5 = strongly disagree	1
4, 7, 9, 10, 12, 13, 14, 16, 17	1 = strongly agree	1
	2 = agree	2
	3 = uncertain	3
	4 = disagree	4
	5 = strongly disagree	5

© 2022 Xie L et al. *JAMA Network Open*.
