# Supplementary material for: Association Between Patient Satisfaction With Their Patient-Physician Relationship and Completion of Bariatric Surgery by Race and Ethnicity Among US Adults
Source: JAMA Netw Open. 2022 Dec 19;5(12):e2247431. doi: 10.1001/jamanetworkopen.2022.47431 (PMC9856898; doi:10.1001/jamanetworkopen.2022.47431)
Supplement: Supplement 2. — Data Sharing Statement [file jamanetwopen-e2247431-s002.pdf]

Data Sharing Statement

Xie. Association Between Patient Satisfaction With Their Patient-Physician Relationship and Completion of Bariatric Surgery by Race and Ethnicity Among US Adults. *JAMA Netw Open*. Published December 19, 2022. doi:10.1001/jamanetworkopen.2022.47431

Data
Data available: No
